# Diagnosis of chlamydial and gonococcal conjunctivitis: performance evaluation of a nucleic acid amplification test

**DOI:** 10.1128/spectrum.00701-26

**Published:** 2026-07-10

**Authors:** Olivier Bédard, Émilie Vallières, Julie Vadboncoeur, Patrick Hamel, Christian Lavallée, Brigitte Lefebvre, Judith Fafard, Simon Lévesque, Mariam Ag Bazet, Claude Fortin, Annie-Claude Labbé

**Affiliations:** 1 Faculty of Medicine, Université de Montréal, Montréal, Canada; 2 Division of Microbiology, Department of Clinical Laboratory Medicine, Centre Hospitalier Universitaire Sainte-Justine, Montréal, Canada; 3 Division of Pediatric Infectious Diseases and Microbiology, Centre Hospitalier Universitaire Sainte-Justine, Montréal, Canada; 4 Department of Microbiology, Infectious Diseases, and Immunology, Université de Montréal, Montréal, Canada; 5 Department of Ophthalmology, Université de Montréal, Montréal, Canada; 6 Centre Universitaire d’Ophtalmologie (CUO), Hôpital Maisonneuve-Rosemont, CIUSSS de l’Est-de-l’île-de-Montréal, Montréal, Canada; 7 Division of Ophthalmology, Centre Hospitalier Universitaire Sainte-Justine, Montréal, Canada; 8 Division of Infectious Diseases and Medical Microbiology, Hôpital Maisonneuve-Rosemont, CIUSSS de l’Est-de-l’île-de-Montréal, Montréal, Canada; 9 Division of Microbiology, Department of Clinical Laboratory Medicine, Centre Hospitalier de l’Université de Montréal, Montréal, Canada; 10 Laboratoire de santé publique du Québec, Institut national de santé publique du Québec, Ste-Anne-de-Bellevue, Canada; 11 Division of Infectious Diseases and Microbiology, Centre Hospitalier Universitaire de Sherbrooke, CIUSSS de l’Estrie, Sherbrooke, Canada; 12 Department of Microbiology and Infectious Diseases, Faculty of Medicine and Health Sciences, Université de Sherbrooke, Sherbrooke, Canada; 13 Division of Infectious Diseases, Centre hospitalier de l'Université de Montréal, Montréal, Canada; ARUP Laboratories, Salt Lake City, Utah, USA

**Keywords:** validation, NAAT, *Neisseria gonorrhoeae*, *Chlamydia trachomatis*, conjunctivitis

## Abstract

No regulatory agency-approved nucleic acid amplification tests (NAATs) exist for detecting *Chlamydia trachomatis* (CT) or *Neisseria gonorrhoeae* (NG) in conjunctival samples, necessitating validation, including performance analysis. This is challenging due to case rarity and the absence of a gold standard. This retrospective case series evaluated the performance of the Roche cobas CT/NG NAAT on conjunctival samples using microbiological and clinical data. Clinical records of patients with positive CT/NG NAAT conjunctival samples processed in our laboratory between 2013 and 2023 were reviewed, along with matched negative controls. Positive results were confirmed using a stepwise algorithm: (i) positive NG culture (NG only); (ii) confirmatory CT/NG NAAT performed at the Laboratoire de santé publique du Québec (LSPQ); (iii) CT/NG NAAT from other anatomical sites; or (iv) clinical presentation compatible with CT/NG conjunctivitis. Negative controls were confirmed based on clinical information. Sixty-five positive conjunctival samples were included (53 CT-positive, 12 NG-positive). Of the 53 CT-positive samples, 52 were confirmed (21 by LSPQ testing, five by a concomitant genital sample, and 26 by a compatible presentation). Of 53 CT-negative controls, 45 were clinically confirmed as true negatives. Among the 12 NG-positive samples, 11 were confirmed (seven by culture, four by LSPQ testing). Of 12 NG-negative controls, 11 were clinically confirmed. Sensitivity and specificity were 87% and 98% for CT, and both were 92% for NG. Given sufficient data, the Roche cobas CT NAAT assay on conjunctival samples does not require confirmatory testing. However, confirmation of NG-positive results should continue until more data are available.

**IMPORTANCE:**

*Chlamydia trachomatis* (CT) conjunctivitis has been increasingly observed in adults and neonates. If untreated, it can cause local complications and, in adults, contribute to ongoing transmission. *Neisseria gonorrhoeae* (NG) conjunctivitis is rarer, but its hyperacute presentation can lead to severe outcomes, such as orbital cellulitis or globe rupture. Conventional diagnostic methods for CT are either unavailable or highly resource-consuming, and NG culture lacks sensitivity, underscoring the need for rapid and reliable diagnostic methods. Nucleic acid amplification tests (NAATs) are considered the gold standard for detecting CT and NG in urogenital, pharyngeal, and rectal sites, due to their high sensitivity and specificity. Despite widespread use on conjunctival specimens, no NAAT is approved by regulatory agencies. Evaluation of the Roche cobas CT/NG assay is crucial to establish its performance on conjunctival specimens and to provide clinicians with accurate, timely diagnostic results.

## INTRODUCTION

Chlamydial and gonococcal infections remain a growing concern (1). The rising infection rate among adults has resulted in multiple consequences, including a theoretical upsurge in vertical transmission to newborns, as well as an increase in extragenital infections, including conjunctivitis, which can affect both adults and newborns (2–5).

*Chlamydia trachomatis* (CT) conjunctivitis, distinct from trachoma, is a rare clinical manifestation but has been increasingly diagnosed in recent years. Infection usually occurs after inoculation with genital secretions from an infected individual, most often from the individuals themselves (6). Concomitant urogenital involvement is common, but frequently asymptomatic. Chlamydial conjunctivitis is described as a chronic unilateral follicular conjunctivitis, affecting the lower palpebral conjunctiva. Most often, patients complain of mild symptoms such as redness, light pain, or foreign body sensation evolving from weeks to months. Even when the presentation is acute, symptoms are usually not debilitating. It may also present, particularly in neonates, with either purulent or blood-stained discharge (6, 7). Infections are most common in sexually active individuals and in neonates.

Gonococcal conjunctivitis is even rarer and typically presents similarly in adults and neonates, as hyperacute, with conjunctival injection and copious mucopurulent discharge (5, 8). It can lead to significant corneal involvement, including rapid progression to ulceration, perforation, and, in rare severe cases, ocular globe rupture (6, 9).

Before nucleic acid amplification tests (NAATs) became available to diagnose CT infections, other diagnostic methods, such as Giemsa stain, immunofluorescence, and cell culture, were used (10, 11). These methods were time-consuming and required significant specialized resources. Aside from NAATs, the only other diagnostic method for NG is agar culture, which is known for its low sensitivity (~50%), especially in extragenital samples (12).

NAATs are fast, easy, and low-cost diagnostic methods for both CT and NG. Commercial assays have been developed and approved by regulatory agencies only for use on urine, vaginal, cervical, rectal, and pharyngeal samples (13, 14). To ensure optimal laboratory practices, these tests can be used on specimens from other anatomical sites only after completion of a validation process, which includes performance analysis (15, 16).

Although many studies have established the clinical relevance of using CT/NG NAATs on conjunctival samples (9, 17), current guidelines from Canada, the United States, Europe, and Australia provide limited guidance on diagnostic methods for chlamydial and gonococcal conjunctivitis (12–14, 18–22). Only one peer-reviewed research paper was published on the performance of a NAAT assay on conjunctival samples for CT, and there have been no studies for NG (23). The CT study was performed using Hologic’s Gen-Probe Aptima Combo 2 CT/NG assay, which is not the assay used in our laboratory. Consequently, a performance evaluation process was necessary to ensure the reliability of the Roche cobas 4800 and 5800/6800/8800 CT/NG assays for conjunctival specimens.

## MATERIALS AND METHODS

### Data collection

A list of all test results for CT/NG NAATs performed on conjunctival samples between January 1, 2013, and December 31, 2024, was generated from the Hôpital Maisonneuve-Rosemont (HMR) Laboratory Information System (LIS). The HMR laboratory is responsible for processing tests from numerous clinics and hospitals, including the HMR and the Center Hospitalier Universitaire Sainte-Justine (CHUSJ), a tertiary mother and child care center.

Conjunctival swabs positive for CT or NG were included in the analysis if the patient from whom the sample was collected had been clinically evaluated at the HMR or at the CHUSJ. A retrospective analysis of medical records was conducted to retrieve demographic information, clinical presentation, and treatment for every CT- or NG-positive case. Samples were excluded from the analysis if no clinical information was available. Matched negative controls were also selected from the LIS-generated list, and their medical records were also reviewed.

### Primary and confirmatory testing

Conjunctival samples were collected by physicians with the Roche woven swab and transported in the cobas PCR Media.

**Fig 1:**
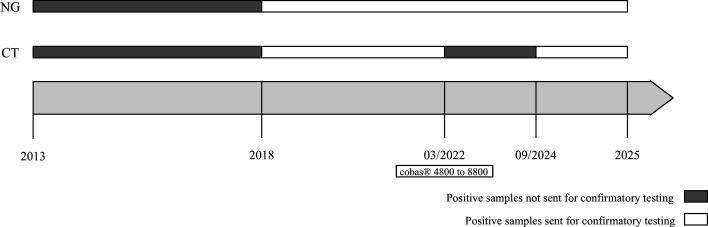
Timeline of confirmatory testing performed at the Laboratoire de santé publique du Québec on conjunctival swabs positive for *Chlamydia trachomatis* or *Neisseria gonorrhoeae.* Primary testing at the Hôpital Maisonneuve-Rosemont (HMR) was performed on the Roche cobas 4800 system until March 2022, with the migration to the Roche cobas 8800 system. CT, *Chlamydia trachomatis*; NG, *Neisseria gonorrhoeae.*

As detailed in Fig. 1, NAATs have been available at the HMR microbiology laboratory since 2012 for the diagnosis of CT/NG conjunctivitis. Testing was performed using the Roche cobas 4800 system from January 2013 until March 2022, when the analysis transitioned to the Roche cobas 8800 system. The assays have different reagent compositions, but primers are the same. They target the cryptic plasmid and chromosomal DNA for CT and the DR-9a and DR-9b region for NG (24). In 2022, before the transition from Roche cobas 4800 to the Roche cobas 8800 analyzer, a method comparison was undertaken to assess the results concordance between the Roche cobas 4800 assay and the Roche cobas 5800/6800/8800 CT/NG assay. This evaluation was done using 193 samples, including 21 from conjunctival swabs (9 CT+/NG−, 3 NG+/CT−, and 9 CT−/NG); concordance was 100%. In addition, internal and external reproducibility with the Roche cobas 5800/6800/8800 CT/NG assay was evaluated on different sample types, including conjunctival samples (*n* = 11), with identical results across repetitions (data not shown).

Since 2018, positive samples for CT/NG were sent to the Laboratoire de santé publique du Québec (LSPQ; provincial reference laboratory) for secondary testing, with brief interruption for CT for logistical reasons. Between 2013 and 2018, secondary testing was not done, neither for CT nor for NG. The CT NAAT available at the LSPQ was originally developed for genotyping CT positive samples rather than for confirmatory purposes. In the present study, however, the LSPQ CT NAAT was considered a confirmatory method, as it detects different gene targets than the Roche cobas assay. It is a real-time PCR targeting the cryptic plasmid and *pmpH* gene (25). In contrast, the NG NAAT available at the LSPQ was originally designed as a confirmatory test and is a duplex real-time PCR targeting the *opa* and *porA* genes (26). The primers used for both confirmatory tests have different genetic targets (cryptic plasmid and *pmpH* for CT; *porA* and *opa* genes for NG) than those in the Roche cobas NAAT assays.

### Definition of confirmed positive

**Fig 2:**
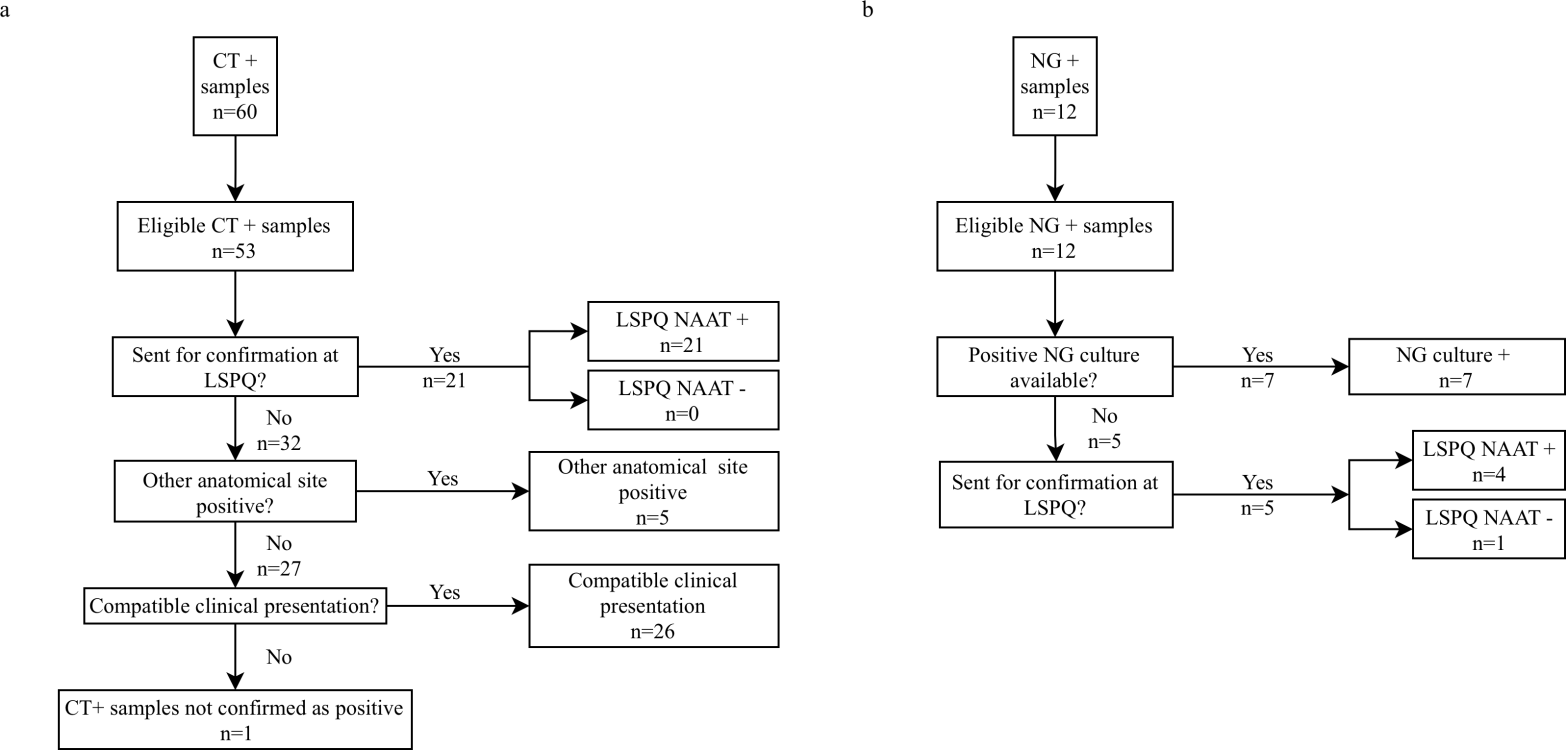
Flow charts of confirmation methods of positive conjunctival samples for *Chlamydia trachomatis* and *Neisseria gonorrhoeae*. (**a**) For *Chlamydia trachomatis* (CT), 52/53 eligible positive NAATs were confirmed as positive. (**b**) For *Neisseria gonorrhoeae* (NG), 11/12 eligible positive NAATs were confirmed as positive. Compatible clinical presentation: clinical presentation, other diagnostic test results, prescribed treatment, and response to that treatment were evaluated by ophthalmologists and infectious disease specialists. This comprehensive clinical assessment was then used to determine whether the findings were compatible or incompatible with CT infection, thereby confirming or invalidating the Roche cobas NAAT result.

Due to changes in confirmation strategies between 2013 and 2024, positive conjunctival NAATs were confirmed using a stepwise approach (Fig. 2): (i) positive NG culture (NG only); (ii) confirmatory LSPQ NAAT (positive result confirmed true positive, negative result indicated false positive); (iii) concomitant (+/− 14 days from the conjunctival NAAT collection date) positive NAAT from another anatomical site (urine, cervix, vagina, or pharynx); and (iv) clinical compatibility assessment from the patients’ medical records (presentation, diagnostic findings, treatment prescribed, and treatment response).

### Controls

Each positive conjunctival sample for CT/NG (case) was matched to a negative control. Controls were selected in chronological order by identifying the next negative CT/NG result obtained from a conjunctival specimen collected from the same hospital. This was done to control for potential pre-analytical or medical record differences between the HMR and the CHUSJ, and to ensure that positive and negative samples were analyzed with the same kind of information.

### Chart review and definitions used

Clinical chart review was done for all cases and controls. Information on clinical presentation, other diagnostic tests performed, treatment prescribed, and response to treatment was extracted. Patients with no clinical record available were excluded, and another negative control was identified. Negative samples were confirmed as true negatives if the clinical presentation was incompatible with CT or NG conjunctivitis; if an alternate diagnosis was confirmed microbiologically, clinically, or pathologically; if there was no improvement following adequate treatment for CT or NG; or if symptoms resolved without adequate treatment for CT or NG. Negative samples were not confirmed if none of the aforementioned criteria were fulfilled.

A compatible clinical presentation for CT conjunctivitis is defined as a chronic, unilateral, or bilateral follicular conjunctivitis. Improvement after the administration of standard systemic antibiotics for CT strongly suggests a diagnosis of chlamydial conjunctivitis. In neonates, hemorrhagic conjunctivitis was also considered as a compatible presentation. When available, the presence of risk factors for sexually transmitted infections was taken into consideration for adults.

A compatible clinical presentation for NG conjunctivitis is defined as an acute or hyperacute unilateral mucopurulent conjunctivitis or keratitis. Gonococcal conjunctivitis can also present as pre-septal or orbital cellulitis. Improvement after the administration of usual systemic antibiotics for NG is strongly suggestive of gonococcal conjunctivitis.

Negative control samples were considered true negatives if the clinical presentation was incompatible with CT or NG conjunctivitis, if an alternate diagnosis was confirmed (microbiologically, clinically, or pathologically), if there was no improvement following adequate treatment for CT or NG, or if symptoms resolved without administration of systemic treatment that could potentially cure CT/NG conjunctivitis, since this infection is not known to be self-limiting (27). Negative control samples were not confirmed if none of the aforementioned criteria were fulfilled and were considered as “potentially compatible presentation.” If not enough information was available to confirm, the control sample was considered “indeterminate.” Both “potentially compatible” and “indeterminate” presentations were conservatively tabulated as false negatives for analytical purposes.

### Statistical analysis

Each sample was tabulated as a true positive, false positive, true negative, or false negative result. Sensitivity, specificity, positive predictive value (PPV), and negative predictive value (NPV) were calculated with their 95% confidence intervals (CIs). Upon medical record review, negative Roche cobas NAATs that were classified as “indeterminate” were conservatively considered as false negatives.

## RESULTS

### Number of conjunctival samples tested and positivity rate

**Fig 3:**
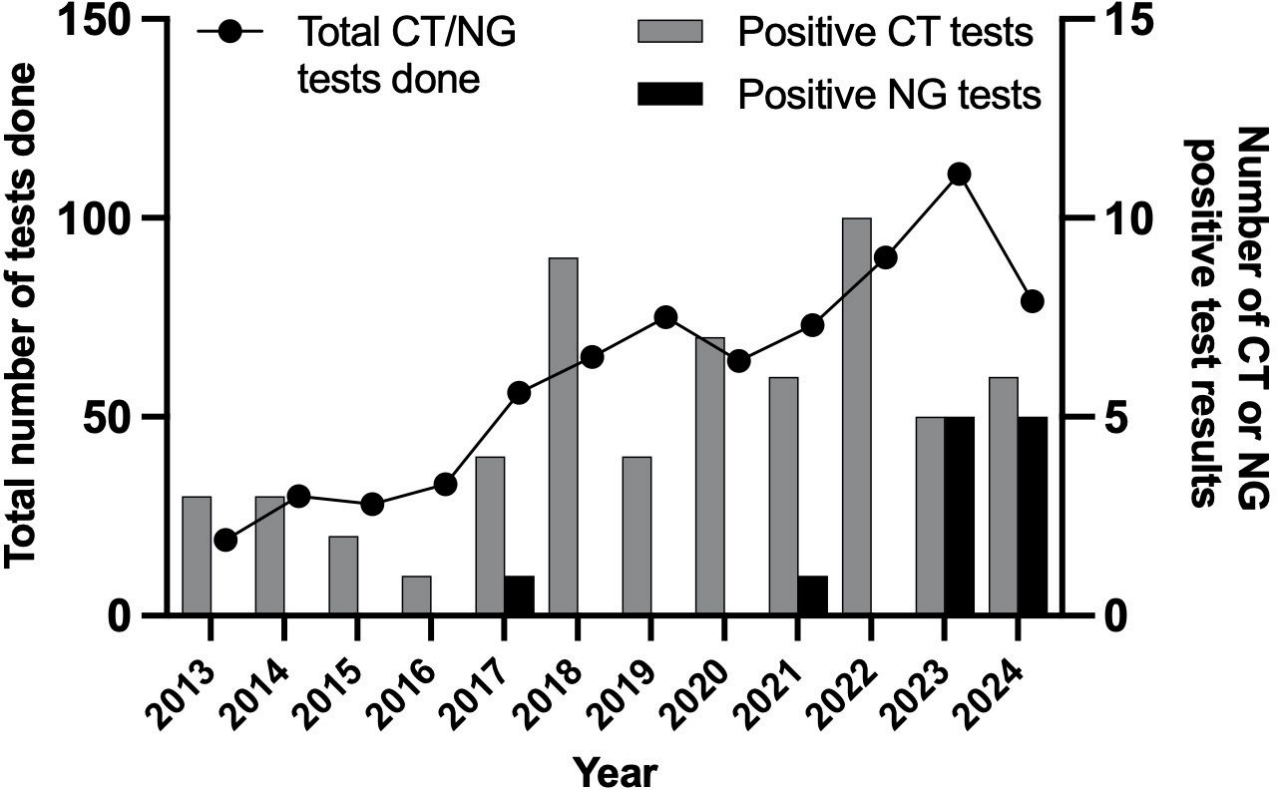
Total number of CT/NG NAATs and number of positive NAATs for CT and NG on conjunctival samples between 2013 and 2024. The total number of CT/NG NAATs performed annually at the HMR is depicted by the line (left y-axis), and the number of positive NAATs for CT (gray) and NG (black) is depicted by the bars (right y-axis). CT, *Chlamydia trachomatis*; NG, *Neisseria gonorrhoeae*.

Over the study period, 723 conjunctival samples were tested for CT/NG using the Roche cobas CT/NG NAAT assay (Fig. 3). The yearly number of samples increased from 19 in 2013 to 111 in 2023, before declining to 79 in 2024. Overall, 60 samples (8.3%) were positive for CT, and 12 were positive for NG (1.7%). The majority (*n* = 10, 83.3%) of NG-positive samples were identified in 2023 and 2024. Two samples, from the same individual, were positive for both CT and NG.

### *Chlamydia trachomatis* conjunctival samples

Among the 60 CT-positive conjunctival samples, seven were excluded due to the unavailability of associated clinical records, yielding 53 eligible CT-positive samples collected from 40 distinct individuals. Some individuals had more than one positive sample because of bilateral involvement (nine individuals) or repeated sampling (three individuals). A NAAT result was available from the LSPQ for 21/53 (39,6%) samples; all of them were positive. Among the remaining 32 eligible CT-positive samples, five had CT-positive results obtained from other anatomical sites sampled concomitantly (urine = 2, vaginal swab = 2, and cervical swab = 1). The remaining 27 CT-positive samples were collected from 21 individuals. As detailed in the Table S1, the clinical presentation was considered compatible with chlamydial conjunctivitis in 20 patients (26 samples). The remaining sample was from an immunocompromised individual with ocular graft-versus-host disease who received incomplete treatment with azithromycin and whose conjunctival culture was positive for *S. aureus*. Given those confounding factors, it was conservatively classified as a potential false positive, even if the clinical presentation appeared compatible with chlamydial conjunctivitis (Table S1; individual pCT-17).

A chart review of the 53 selected patients who had a CT-negative NAAT (negative controls) showed that 45 had a non-compatible clinical presentation for chlamydial conjunctivitis, while eight had a potentially compatible or indeterminate presentation. More information about each case and the rationale supporting the assessment of presentation compatibility is available in Table S2.

From these findings, as detailed in Table 1, the Roche cobas CT NAAT was calculated to have a sensitivity of 86.6% (95% CI [75.4–94.1]) and a specificity of 97.8% (95% CI [88.5–99.9]). The PPV was 98.1% (95% CI [89.9–100]), and the NPV was 84.9% (95% CI [72.4–93.3]).

### *Neisseria gonorrhoeae* conjunctival samples

Twelve NG-positive conjunctival samples were identified from eight individuals. All were eligible for data analysis. NG culture was performed for all eight patients, and four (50%) were positive. Because some patients had more than one sample tested by NAAT, these four positive NG cultures (in four distinct individuals) represent 7/12 positive NAAT samples. LSPQ confirmatory NAATs were available for the five remaining specimens: four were positive, and one was negative. The clinical presentation of the patient with discordant results, combined with a negative NG culture and a blood culture positive for *Haemophilus influenzae*, was not compatible with gonococcal conjunctivitis.

A chart review of the 12 negative controls for NG NAAT revealed that 11 patients had a non-compatible clinical presentation for gonococcal conjunctivitis and one had a potentially compatible presentation (Table S3).

From these findings, as detailed in Table 1, the Roche cobas NG NAAT was calculated to have a sensitivity of 91.6% (95% CI [61.5, 99.8]) and a specificity of 91.6% (95% CI [61.5, 99.8]). The PPV was 91.6% (95% CI [61.5, 99.8]), and the NPV was 91.6% (95% CI [61.5, 99.8]).

**TABLE 1:** Performance of the Roche cobas nucleic acid amplification test for *Chlamydia trachomatis* and *Neisseria gonorrhoeae* on conjunctival samples^*a*^

	Confirmatory strategies	
	Positive	Negative
*Chlamydia trachomatis*		
Roche cobas CT NAAT result		
Positive	52	1
Negative	8^*b*^	45
*Neisseria gonorrhoeae*		
Roche cobas NG NAAT resut		
Positive	11	1
Negative	1^*c*^	11

^*a*^ CT, *Chlamydia trachomatis*; NAAT, nucleic acid amplification test; NG, *Neisseria gonorrhoeae.*

^*b*^ Of the eight false negative samples, six were from patients with an indeterminate presentation and two from patients with a presentation potentially compatible with chlamydial conjunctivitis.

^*c*^ The false negative sample was from a patient with a presentation potentially compatible with gonococcal conjunctivitis.

## DISCUSSION

This retrospective performance analysis is one of the few studies focusing on NAATs for conjunctival samples (23). Such studies are known to be difficult to conduct due to the rarity of chlamydial and gonococcal conjunctivitis cases. However, the HMR laboratory serves as the regional reference center for CT/NG NAATs on conjunctival samples, which facilitated this effort. Although a full analytical validation was not undertaken, the present study leverages real-world clinical specimens collected over an extended timeframe, thereby offering valuable insight into assay performance in routine practice.

Another challenge with CT/NG assays evaluation is the lack of an adequate gold standard. For CT, all other non-NAATs diagnostic methods are substantially less sensitive than NAATs and have been discontinued in most laboratories. For NG, culture is still available, but is known to have a lower sensitivity, making it an unfit gold standard (28). Moreover, NG culture on conjunctival swabs could potentially be made negative due to widespread empirical use of topical antibiotics for conjunctivitis, which is not the case with NAATs.

For CT, the performance data calculated from our findings align with previous work conducted on other CT NAATs for conjunctival samples (23). Sensitivity and NPV are most likely underestimated in this study, since they are based on data from medical records retrospective review. The CT-negative medical record review was conducted using a conservative approach, classifying as false negatives many patients whose records lacked sufficient clinical evidence to confirm the absence of chlamydial conjunctivitis. If, in a less conservative manner, the “indeterminate” clinical presentations were considered true negatives, the sensitivity, specificity, PPV, and NPV would increase to 96%, 98%, 98%, and 96% respectively.

Limitations include those inherent to retrospective studies: confirmation methods varied during the period of the study, leading to heterogeneous techniques, and the clinical documentation available during the file review was sometimes limited. Elements included in our definitions of compatible clinical presentations were not always systematically recorded. This likely contributed to an underestimation of the performance of the cobas 4800 and 5800/6800/8800 CT/NG assays on conjunctival samples for CT. Nevertheless, enough information was available to confirm good performance of the assay for CT results. This study adds to evidence that NAATs are reliable and should be used for the diagnosis of chlamydial conjunctivitis in clinical settings, given their high sensitivity, specificity, and rapid turnaround time, all of which can directly influence clinical management decisions.

This performance assessment also focused on NG detection by NAAT on conjunctival samples. Only 12 NG-positive samples were available for this study, and one of these was later determined to be a false positive. The wide 95% CIs calculated for sensitivity, specificity, PPV, and NPV are explained by the small number of samples available for our study. Although NAATs are likely reliable for diagnosing gonococcal conjunctivitis, insufficient data are available to confirm this conclusively. The presence of a false positive test in a small sample size raises questions but does not hinder future validation of the assay. The risks of false positive results in other sampling sites, such as the pharynx, have been thoroughly described in the literature. The main explanation for higher false positive rates in pharyngeal swabs is cross-reactivity with other commensal *Neisseria* spp. that are part of the normal flora (29, 30). Although not specifically reported as being part of the normal conjunctival flora, a similar phenomenon could still possibly be observed in conjunctival specimens, given the anatomical proximity to the pharynx (31, 32).

The calculated sensitivity and specificity of the cobas 4800 and 5800/6800/8800 CT/NG NAAT assays on conjunctival samples remain high for NG. This adds to the evidence that NAATs should be considered to diagnose gonococcal conjunctivitis. After excluding the patient with the false positive result (whose NG culture was negative), culture results were positive for four out of the seven patients (57%) with positive NG NAAT. These findings are in line with previous work that described a low sensitivity of culture for detecting NG in extragenital sites (12). Even with the wide 95% CI, the Roche cobas NG NAAT still outperforms culture for conjunctival samples. Also, given the hyperacute presentation associated with gonococcal conjunctivitis, the ability of NAATs to provide timely results is useful for clinical management. Nevertheless, results should be interpreted with caution. We consider that confirmatory testing, such as NG culture and NAATs, remains necessary for the time being. Given the recent rise of NG-positive NAATs observed at the HMR laboratory, it is expected that a more extensive evaluation will eventually be possible in the coming years.

For both CT and NG, false positives could have occurred at the pre-analytical level. Sample collection was performed by various clinicians. Clerical errors or suboptimal swabbing cannot be excluded. False negative results are always possible, particularly when small amounts of genetic material are present, particularly at the very beginning or at the end of an infection episode. These limitations exist with all tests and are not specific to the Roche cobas CT/NG NAAT. Since all CT-negative and NG-negative samples included in our study required comprehensive medical record review, only a small proportion of the negative samples obtained over the entire study period were analyzed. This could influence the specificity and NPV calculations of each test. Also, the retrospective nature of the study limited access to cycle threshold (Ct) values, precluding more detailed analysis of discrepant results. This should be considered when interpreting the findings.

Overall, the Roche cobas CT/NG NAAT demonstrated strong performance for the detection of CT and promising results for NG on conjunctival samples in a real-world clinical setting. These findings support the use of NAATs for the diagnosis of conjunctivitis when CT or NG is suspected, while highlighting the need for continued data accumulation and confirmatory strategies, particularly for NG. Further work, including studies using contrived specimens and reference materials, will be important to more comprehensively define the performance characteristics and complete a full analytical validation of these assays.

## Data Availability

Data are provided within the manuscript or Supplemental material. The data set for the current study is available from the corresponding author on reasonable request.

## SUPPLEMENTAL MATERIAL

Table S1 spectrum.00701-26-s0001.pdf Clinical description for CT-positive samples.

Table S2 spectrum.00701-26-s0002.pdf Clinical description for CT-negative samples.

Table S3 spectrum.00701-26-s0003.pdf Clinical description for NG-negative samples.

ASM does not own the copyrights to Supplemental Material that may be linked to, or accessed through, an article. The authors have granted ASM a non-exclusive, world-wide license to publish the Supplemental Material files. Please contact the corresponding author directly for reuse.

## References

[1] Tuddenham S, Hamill MM, Ghanem KG. 2022. Diagnosis and treatment of sexually transmitted infections: a review. JAMA 327:161–172. doi:10.1001/jama.2021.23487 35015033

[2] Harvey-Lavoie S, Apelian H, Labbé AC, Cox J, Messier-Peet M, Moodie EEM, Fourmigue A, Moore D, Lachowsky NJ, Grace D, Hart TA, Jollimore J, Fortin C, Lambert G. 2021. Community-based prevalence estimates of *Chlamydia trachomatis* and *Neisseria gonorrhoeae* infections among gay, bisexual, and other men who have sex with men in Montréal, Canada. Sex Transm Dis 48:939–944. doi:10.1097/OLQ.0000000000001486 34030155

[3] US Preventive Services Task Force, Curry SJ, Krist AH, Owens DK, Barry MJ, Caughey AB, Davidson KW, Doubeni CA, Kubik M, Landefeld CS, Mangione CM, Silverstein M, Simon MA, Tseng CW, Wong JB. 2019. Ocular Prophylaxis for Gonococcal Ophthalmia Neonatorum: US Preventive Services Task Force Reaffirmation Recommendation Statement. JAMA 321:394–398. doi:10.1001/jama.2018.21367 30694327

[4] Public Health Agency of Canada. 2023. Recommendations on Screening for Chlamydia Trachomatis and Neisseria Gonorrhoeae in Pregnancy, on Government of Canada. Available from: https://www.canada.ca/en/public-health/services/infectious-diseases/sexual-health-sexually-transmitted-infections/canadian-guidelines/national-advisory-committee-stbbi/statements/recommendations-screening-chlamydia-trachomatis-neisseria-gonorrhoeae-pregnancy.html. Retrieved 28 Apr 2026.

[5] American Academy of Ophthalmology. 2025. Use of Erythromycin in the Prevention of Ophthalmia Neonatorum - 2025, on AAO. Available from: https://www.aao.org/education/clinical-statement/use-of-erythromycin-in-prevention-of-neonatal-opht. Retrieved 28 Apr 2026.

[6] Durand ML, Barshak MB, Sobrin L. 2023. Eye infections. N Engl J Med 389:2363–2375. doi:10.1056/NEJMra2216081 38118024

[7] Honkila M, Renko M, Pokka T, Wikström E, Uhari M, Tapiainen T. 2018. Symptoms, signs and long-term prognosis of vertically transmitted chlamydia trachomatis infections. Pediatr Infect Dis J 37:930–933. doi:10.1097/INF.0000000000001925 29389825

[8] Belga S, Gratrix J, Smyczek P, Bertholet L, Read R, Roelofs K, Singh AE. 2019. Gonococcal conjunctivitis in adults: case report and retrospective review of cases in Alberta, Canada, 2000-2016. Sex Transm Dis 46:47–51. doi:10.1097/OLQ.0000000000000897 30044333

[9] McAnena L, Knowles SJ, Curry A, Cassidy L. 2015. Prevalence of gonococcal conjunctivitis in adults and neonates. Eye (Lond) 29:875–880. doi:10.1038/eye.2015.57 25907207 PMC4506339

[10] Workowski KA, Bachmann LH, Chan PA, Johnston CM, Muzny CA, Park I, Reno H, Zenilman JM, Bolan GA. 2021. Sexually Transmitted Infections Treatment Guidelines, 2021. MMWR Recomm Rep 70:1–187. doi:10.15585/mmwr.rr7004a1 PMC8344968 34292926

[11] Rafiei Tabatabaei S, Afjeiee SA, Fallah F, Tahami Zanjani N, Shiva F, Tavakkoly Fard A, Shamshiri AR, Karimi A. 2012. The use of polymerase chain reaction assay versus cell culture in detecting neonatal chlamydial conjunctivitis. Arch Iran Med 15:171–175. 22369307

[12] Miller JM, Binnicker MJ, Campbell S, Carroll KC, Chapin KC, Gonzalez MD, Harrington A, Jerris RC, Kehl SC, Leal SM Jr, Patel R, Pritt BS, Richter SS, Robinson-Dunn B, Snyder JW, Telford S 3rd, Theel ES, Thomson RB Jr, Weinstein MP, Yao JD. 2024. Guide to Utilization of the Microbiology Laboratory for Diagnosis of Infectious Diseases: 2024 Update by the Infectious Diseases Society of America (IDSA) and the American Society for Microbiology (ASM). Clin Infect Dis:ciae104. doi:10.1093/cid/ciae104 38442248

[13] Centers for Disease Control. 2021. Sexually Transmitted Infections Treatments Guidelines: Gonococcal Infections Among Adolescents and Adults. Available from: https://www.cdc.gov/std/treatment-guidelines/gonorrhea-adults.htm. Retrieved 4 Aug 2025.

[14] Centers for Disease Control. 2021. Sexually Transmitted Infections Treatments Guidelines: Chlamydial Infection Among Adolescents and Adults. Available from: https://www.cdc.gov/std/treatment-guidelines/chlamydia.htm. Retrieved 4 Aug 2025.

[15] Burd EM. 2010. Validation of laboratory-developed molecular assays for infectious diseases. Clin Microbiol Rev 23:550–576. doi:10.1128/CMR.00074-09 20610823 PMC2901657

[16] Wallace PS, MacKay WG. 2013. Quality in the molecular microbiology laboratory. Methods Mol Biol 943:49–79. doi:10.1007/978-1-60327-353-4_3 23104281

[17] Thompson PP, Kowalski RP. 2011. A 13-year retrospective review of polymerase chain reaction testing for infectious agents from ocular samples. Ophthalmology 118:1449–1453. doi:10.1016/j.ophtha.2010.12.004 21367461

[18] Cheung AY, Choi DS, Ahmad S, Amescua G, Jhanji V, Lin A, Mian SI, Rhee MK, Viriya ET, Mah FS, Varu DM. 2024. Conjunctivitis preferred practice pattern. Ophthalmology 131:P134–P204. doi:10.1016/j.ophtha.2023.12.037

[19] Public Health Agency of Canada. 2024. Gonorrhea guide: Screening and diagnostic testing, on Government of Canada. Available from: https://www.canada.ca/en/public-health/services/infectious-diseases/sexual-health-sexually-transmitted-infections/canadian-guidelines/gonorrhea/screening-diagnostic-testing.html. Retrieved 4 Aug 2025.

[20] Public Health Agency of Canada. 2025. Chlamydia and LGV guide: Screening and diagnostic testing, on Government of Canada. Available from: https://www.canada.ca/en/public-health/services/infectious-diseases/sexual-health-sexually-transmitted-infections/canadian-guidelines/chlamydia-lgv/screening-diagnostic-testing.html. Retrieved 4 Aug 2025.

[21] White JA, Dukers-Muijrers NH, Hoebe CJ, Kenyon CR, Dc Ross J, Unemo M. 2025. 2025 European guideline on the management of *Chlamydia trachomatis* infections. Int J STD AIDS 36:434–449. doi:10.1177/09564624251323678 40037375

[22] Unemo M, Ross J, Serwin AB, Gomberg M, Cusini M, Jensen JS. 2020. European guideline for the diagnosis and treatment of gonorrhoea in adults. Int J STD AIDS. doi:10.1177/0956462420949126:956462420949126 33121366

[23] Kowalski RP, Karenchak LM, Raju LV, Ismail N. 2015. The verification of nucleic acid amplification testing (Gen-Probe Aptima Assay) for chlamydia trachomatis from ocular samples. Ophthalmology 122:244–247. doi:10.1016/j.ophtha.2014.08.038 25439605

[24] Centers for Disease Control Prevention. 2014. Recommendations for the laboratory-based detection of Chlamydia trachomatis and Neisseria gonorrhoeae--2014. MMWR Recomm Rep 63:1–19. PMC4047970 24622331

[25] Chen CY, Chi KH, Alexander S, Ison CA, Ballard RC. 2008. A real-time quadriplex PCR assay for the diagnosis of rectal lymphogranuloma venereum and non-lymphogranuloma venereum *Chlamydia trachomatis* infections. Sex Transm Infect 84:273–276. doi:10.1136/sti.2007.029058 18283094

[26] Goire N, Nissen MD, LeCornec GM, Sloots TP, Whiley DM. 2008. A duplex *Neisseria gonorrhoeae* real-time polymerase chain reaction assay targeting the gonococcal porA pseudogene and multicopy opa genes. Diagn Microbiol Infect Dis 61:6–12. doi:10.1016/j.diagmicrobio.2007.12.007 18248938

[27] Azari AA, Barney NP. 2013. Conjunctivitis: a systematic review of diagnosis and treatment. JAMA 310:1721–1729. doi:10.1001/jama.2013.280318 24150468 PMC4049531

[28] Nash EE, Pham CD, Raphael B, Learner ER, Mauk K, Weiner J, Mettenbrink C, Thibault CS, Fukuda A, Dobre-Buonya O, Black JM, Johnson K, Sellers K, Schlanger K, Group SW. 2021. Impact of anatomic site, specimen collection timing, and patient symptom status on *Neisseria gonorrhoeae* culture recovery. Sex Transm Dis 48:S151–S156. doi:10.1097/OLQ.0000000000001540 34433797 PMC9125530

[29] Upton A, Bromhead C, Whiley DM. 2013. Neisseria gonorrhoeae false-positive result obtained from a pharyngeal swab by using the Roche cobas 4800 CT/NG assay in New Zealand in 2012. J Clin Microbiol 51:1609–1610. doi:10.1128/JCM.00485-13 23486711 PMC3647902

[30] Tabrizi SN, Unemo M, Limnios AE, Hogan TR, Hjelmevoll SO, Garland SM, Tapsall J. 2011. Evaluation of six commercial nucleic acid amplification tests for detection of Neisseria gonorrhoeae and other Neisseria species. J Clin Microbiol 49:3610–3615. doi:10.1128/JCM.01217-11 21813721 PMC3187337

[31] Huang Y, Yang B, Li W. 2016. Defining the normal core microbiome of conjunctival microbial communities. Clin Microbiol Infect 22:643. doi:10.1016/j.cmi.2016.04.008 27102141

[32] Dong Q, Brulc JM, Iovieno A, Bates B, Garoutte A, Miller D, Revanna KV, Gao X, Antonopoulos DA, Slepak VZ, Shestopalov VI. 2011. Diversity of bacteria at healthy human conjunctiva. Invest Ophthalmol Vis Sci 52:5408–5413. doi:10.1167/iovs.10-6939 21571682 PMC3176057

