# Supplementary material for: Diagnosis of chlamydial and gonococcal conjunctivitis: performance evaluation of a nucleic acid amplification test
Source: Microbiol Spectr. 2026 Jul 10;14(8):e00701-26. doi: 10.1128/spectrum.00701-26 (PMC13436316; doi:10.1128/spectrum.00701-26)
Supplement: Supplementary file 1 [file spectrum.00701-26-s0001.pdf]

Table S1 - Clinical description for CT-positive samples

#	Number of samples	Brief clinical description	Decision	Rationale (if compatible)
pCT-01	1	Adult male - Chronic unilateral follicular conjunctivitis. Empirical topical treatment: tobramycin and corticosteroids. No improvement. No follow up or prescription available after diagnosis of CT.	Compatible	Typical presentation for CT
pCT-02	2	Newborn female - Acute bilateral purulent conjunctivitis with blood-stained discharge. Treated with topical erythromycin without improvement. Given oral erythromycin. Complete resolution of symptoms at follow up.	Compatible	Typical presentation for CT / Improvement with CT treatment
pCT-03	1	Adult male - Chronic unilateral follicular conjunctivitis. Treated with topical antibiotics and artificial tears without improvement. Treated with doxycyclin. Complete resolution of symptoms at follow up.	Compatible	Typical presentation for CT / Improvement with CT treatment
pCT-04	1	Adult male - Chronic unilateral follicular conjunctivitis. Multiple male sexual partners. Treated with topical antibiotics without improvement. Prescribed azithromycin, complete resolution at follow up.	Compatible	Typical presentation for CT / Improvement with CT treatment
pCT-05	1	Adult male - Chronic bilateral follicular conjunctivitis. Topical antibiotics prescribed without improvement. Prescribed doxycyclin, complete resolution at follow up.	Compatible	Typical presentation for CT / Improvement with CT treatment
pCT-06	1	Adult male - Past medical history of ocular trauma, followed by ophtalmologist. Chronic unilateral follicular conjunctivitis, no discharge. Had 2 or 3 sexual partners in the previous year. Treated with azithromycin; resolution of conjunctivtis thereafter.	Compatible	Typical presentation for CT / Resolution with CT treatment
pCT-07	1	Adult male - Presented with chronic bilateral chronic follicular conjunctivitis. Was prescribed systemic azithromycin. No follow up available.	Compatible	Typical presentation for CT
pCT-08	1	Adult male - Presented with chronic unilateral chronic follicular conjunctivitis. Was prescribed systemic azithromycin. No follow up available.	Compatible	Typical presentation for CT
pCT-09	1	Adult male - Chronic unilateral follicular conjunctivitis with slight purulent discharge. Had an unprotected sexual intercourse 4 months prior. Treated with doxycyclin and topical erythromycin. Symptoms improved with treatment. Residual follicles treated with fluorometholone. No more follow up.	Compatible	Typical presentation for CT / Partial resolution with CT treatment
pCT-10	2	Adult female - Acute bilateral follicular conjunctivitis with mucopurulent discharge. Treated with azithromycin empirically, along with partner. NAAT positive from both eyes. Symptoms resolved within 10 days. Councelled to see gynecologist for examination (not available).	Compatible	Typical presentation for CT / Resolution with CT treatment

pCT-11	2	Adult male - Chronic unilateral follicular conjunctivitis with mucopurulent discharge. Treated with topical antibiotics without improvement. Recent history of untreated urethritis (urine tests not available). Conjunctival CT NAAT positive twice. Treated with both doxycyclin and azithromycin. Complete resolution of symptoms at follow up.	Compatible	Typical presentation for CT / Resolution with CT treatment
pCT-12	1	Adult female - Subacute unilateral follicular conjunctivitis with subepithelial infiltrate. Topical tobramycin prescribed with no improvement. New sexual partner for the past 5 months. Treated with azythromycin. No follow up available. Refered for STI screening.	Compatible	Typical presentation for CT
pCT-13	2	Adult female - Acute bilateral follicular conjunctivitis with mucopurulent discharge. Treated with topical antibiotics without improvement. CT NAAT positive from both eyes. Patient and partner treated with azithromycin, complete resolution of symptoms at follow up.	Compatible	Typical presentation for CT / Resolution with CT treatment
pCT-14	1	Adult male - Chronic unilateral follicular conjunctivitis with subepithelial infiltrate. Topical antibiotics and corticosteroids prescribed with no improvement. Patient did not show up for follow up, no treatment prescribed.	Compatible	Typical presentation for CT
pCT-15	2	Adult male - Acute bilateral follicular conjunctivitis with slight mucopurulent discharge. Topical antibiotics and corticosteroids prescribed with no improvement. CT NAAT positive from both eyes. Partner tested positive for CT the week before. Treated with azithromycin, no follow up available.	Compatible	Typical presentation for CT / Positive epidemiological contact
pCT-16	1	Adult male - Chronic unilateral follicular conjunctivitis. Treated empirically with azithromycin. Complete resolution of symptoms at follow up.	Compatible	Typical presentation for CT / Resolution with CT treatment
pCT-17	1	Adult male - Past medical history of hematopoietic stem cell transplant with ocular graft vs host disease (GVHD). Acute follicular unilateral conjunctivitis with mucopurulent discharge and pseudomembranes. At presentation, complaints of urethritis symptoms (not tested). Treated with topical moxifloxacin. Hospitalized the following week for hepatitis and sinusitis. Conjunctival culture positive for *Staphylococcus aureus*. During hospitalization, received azithromycin 500 mg once, piperacilin-tazobactam, and amoxicillin-clavulanate. Resolution at follow up 1 month later.	Indeterminate	

pCT-18	1	Adult female - Chronic unilateral follicular conjunctivitis. Prescribed many topical antibiotics and corticosteroids without improvement. Treated empirically with azithromycin. Complete resolution of symptoms at follow up.	Compatible	Typical presentation for CT / Resolution with CT treatment
pCT-19	1	Adult female - Acute unilateral follicular conjunctivitis. Lubricating drops with no improvement. Treated empirically with azithromycin at follow up. No other follow up.	Compatible	Typical presentation for CT
pCT-20	2	Adult male - Acute bilateral follicular conjunctivitis with slight discharge. Treated with lubricating drops with no improvement. Patient asks for empirical treatment for STIs since he considers himself at risk (no more details). Treated with ceftriaxone and doxycyclin. CT NAAT positive from both eyes. Resolution of symptoms at follow up.	Compatible	Typical presentation for CT / Resolution with CT treatment
pCT-21	1	Adult male - Acute bilateral follicular conjunctivitis. Treated with topical antibiotics with no improvement. Prescribed doxycycline since other sites apparently positive (not available). Improvement of symptoms at follow up.	Compatible	Typical presentation for CT / Improvement with CT treatment

#: arbitrary numbering system; pCT: positive for *Chlamydia trachomatis* (CT); CT: *Chlamydia trachomatis;* NAAT: Nucleic acid amplification test; STI: Sexually transmitted infection
