# Supplementary material for: Diagnosis of chlamydial and gonococcal conjunctivitis: performance evaluation of a nucleic acid amplification test
Source: Microbiol Spectr. 2026 Jul 10;14(8):e00701-26. doi: 10.1128/spectrum.00701-26 (PMC13436316; doi:10.1128/spectrum.00701-26)
Supplement: Supplementary file 2 [file spectrum.00701-26-s0002.pdf]

Table S2 - Clinical description for CT-negative samples

ID	Brief clinical description	Decision	Rationale if not compatible
nCT-01	Adult male - Dry eyes with corneal erosion secondary to acnea treatment. Improvement with tear duct plugs, artificial tears and loteprenol drops.	Not compatible	Improvement without treatment for CT
nCT-02	Adult female - Cyclical follicular and papillar conjunctivitis secondary to allergies. Treated with artificial tears and bepotastin.	Not compatible	Improvement without treatment for CT
nCT-03	Adult male - Chronic unilateral papillar/follicular conjunctivitis for one year. Viral culture negative. Tobramycin/dexamethasone drops prescribed. No follow up.	Indeterminate	
nCT-04	Newborn - Bilateral yellow crusts. Conjuncival bacterial culture positive for S. mitis/oralis. Resolution of symptoms with topical erythromycin.	Not compatible	Alternative diagnosis available
nCT-05	Newborn female - Acute conjunctivitis with pre-septal cellulitis, following an episode of URTI. Initially presented as mucopurulent discharge. Admitted to hospital, treated with IV cefuroxime and topical erythromycin. Complete resolution within 7 days of treatment. Blood culture / conjunctival culture negative.	Not compatible	Not typical presentation for CT / Improvement without treatment for CT
nCT-06	Adult male - Testicular mass with positive urinalysis. Urethritis and conjunctivitis at the same time. Urine CT NAAT result was indeterminate. History of possible foreing objets in eye. Treated with ceftriaxone/azithromycin empirically, topical erythromycin and ciprofloxacin for 10 days. Testicular ultrasound positive for epididymitis. Patient was not made aware of results, returned 1.5 months later with the same symptoms. Received ciprofloxacin. Complete resolution.	Not compatible	Not typical presentation for CT / No improvement with CT treatment
nCT-07	Adult female - Conjunctival angioma removal, then chronic follicular bilateral conjunctivitis. Dry eyes. Prescription of artificial tears. Resolution.	Not compatible	Not typical presentation for CT / Improvement without treatment for CT
nCT-08	Adult female - Chronic unilateral conjunctivitis. Topical corticosteroids prescribed and patient felt slightly better. Final diagnosis was lower eyelid papilloma which was removed; conjunctivitis resolved afterwards.	Not compatible	Improvement without treatment for CT / Alternative diagnosis available
nCT-09	Adult male - Chronic unilateral conjunctivitis. Treated with corticosteroids, with complete resolution.	Not compatible	Improvement without treatment for CT
nCT-10	Adult male - Excessive tearing for the past 2 years. Follicular conjunctivitis on dry eyes. Symptoms resolution with lubricant drops.	Not compatible	Improvement without treatment for CT

nCT-11	Adult female - Chronic unilateral conjunctivitis. Received azithromycin empirically, along with blephamide and TMP-SMX. No follow up.	Indeterminate	
nCT-12	Adult female - Chronic follicular bilateral conjunctivitis, Received azithromycin empirically. No improvement (symptoms worsened). Final diagnosis: allergic conjunctivitis. Topical and systemic anti-histamines resolved the issue.	Not compatible	Not typical presentation for CT /Alternative diagnosis available/ Improvement without treatment for CT
nCT-13	Adult female - Chronic follicular bilateral conjunctivitis. Topical prednisone prescribed. Biopsy: lympocytic infiltrate. Final diagnosis: allergic reaction to the ointment applied by the patient.	Not compatible	Not typical presentation for CT /Alternative diagnosis available/ Improvement without treatment for CT
nCT-14	Adult male - Indeterminate follicular conjunctivitis in a patient with pterygon. Tears prescribed. No follow up.	Indeterminate	
nCT-15	Adult male - Ocular globe trauma with rupture. Prophylactic antibiotics given. No sign of infection.	Not compatible	Not typical presentation for CT /Alternative diagnosis available
nCT-16	Adult male - Presumed viral follicular chronic conjunctivitis. Treated empirically with azythromycin, ceftriaxone and topical erythromycin. No follow up.	Potentially compatible presentation	
nCT-17	Adult female - Chronic follicular conjunctivitis. Conjunctival biopsy: lymphoid hyperplasia, fibrosis.	Not compatible	Alternative diagnosis available Other pathologically proven diagnosis
nCT-18	Adult male - Papillary conjunctivitis in a patient using brimonidine drops. Resolution of conjunctivitis with discontinuation of brimonidine. Final diagnosis: Brimonidine allergy.	Not compatible	Alternative diagnosis available/Improvement without treatment for CT
nCT-19	Adult female - Severe chronic follicular conjunctivitis. CT NAAT was negative on three different occasions. Azithromycin empirically prescribed. Follow-up 1 month later: persistent symptoms. Eyelid hygiene and topical erythromycin prescribed. Slightly better 2 months later.	Not compatible	Not typical presentation for CT / Improvement without treatment for CT / No improvement with CT treatment
nCT-20	Adult female - Papillary chronic conjunctivitis. Biopsy: chronic inflammation. Final diagnosis: dry eyes. Artificial tears prescribed.	Indeterminate	
nCT-21	Adult female - Acute presentation of eyelid swelling, redness and mucopurulent discharge. Diagnosis of pre-septal cellulitis. CT cell culture negative. Ceftriaxone given empirically, then treated with amoxicillin/clavulanic acid for 10 days. Resolution at follow up.	Not compatible	Not typical presentation for CT / Improvement without treatment for CT
nCT-22	Adult female - Unilateral eye redness and discharge for 6 months. Treated twice with empirical azithromycin with no improvement. Final diagnosis: Floppy eyelid syndome with secondary chronic conjunctivitis. Hydrating drops prescribed.	Not compatible	Not typical presentation for CT / Alternative diagnosis available/ No improvement with CT treatment

nCT-23	10 year old male - Conjunctival mass after foreign body removal in eye. Topical tobramycin prescribed without improvement. Final diagnosis: pyogenic granuloma vs hemangioma. Follow up only if still present. No follow up.	Not compatible	Not typical presentation for CT / Alternative diagnosis available / Improvement without treatment for CT
nCT-24	Adult female - Chronic follicular bilateral conjunctivitis for 2 years. Tear duct plugs inserted. MALT lymphoma suspected but biopsy negative. No follow up.	Indeterminate	
nCT-25	Adult male - Blepharitis/chalazion. Chronic unilateral follicular conjunctivitis, then bilateral. Treated with doxycyclin for months without improvement. Repeat CT NAAT negative.	Not compatible	Not typical presentation for CT / No improvement with CT treatment
nCT-26	Adult male - Chronic conjunctivitis. Fibrous changes, multiple conjunctival microabcesses secondary to trichiasis and filamentary keratitis. Bacterial conjunctiva culture positive for *Staphylococcus epidermidis, Cutinebacterium acnes* and Gram positive bacilli.	Not compatible	Not typical presentation for CT
nCT-27	Adult male - Chronic papillary unilateral conjunctivitis with mucopurulent discharge. Diagnosis: floppy eyelid syndrome. Topical tobramycin and prednisolone prescribed with resolution at follow-up.	Not compatible	Not typical presentation for CT / Improvement without treatment for CT
nCT-28	Adult female - Chronic bilateral follicular conjunctivitis with eye pain. Treated with valacyclovir without improvement. No treatment for CT. Final diagnosis: allergic conjunctivitis presumed to be triggered by cats. Resolution with antihistamines.	Not compatible	Improvement without treatment for CT
nCT-29	Adult female - Follicular conjunctivitis, then hemorragic keratoconjunctivitis. Improvement of symptoms with topical prednisone and erythromycin. Household members with similar symptoms.	Not compatible	Not typical presentation for CT / Improvement without treatment for CT
nCT-30	3 year old female - Palpebral nodules with slight discharge for a few months. Final diagnosis: chronic posterior blepharitis with chalazion and conjunctival granulomas.	Not compatible	Not typical presentation for CT
nCT-31	Adult male - Chronic follicular conjunctivitis in a patient with past medical history of ophthalmic zoster and labial/genital HSV. Adenovirus vs HSV suspected. Complete resolution of symptoms with valacyclovir and tobramycin/dexamethasone drops. Conjunctival culture negative.	Not compatible	Not typical presentation for CT / Improvement without treatment for CT

nCT-32	Adult male - Eye redness, decreased visual acuity, ocular pain. Final diagnosis: corneal ulcer which complicated into bullous keratopathy. Culture: *Staphylococcus aureus*.	Not compatible	Not typical presentation for CT / Alternative diagnosis available
nCT-33	Neonate male - Hospitalized for URTI, discharged from hospital. Comes back 2 days later with light redness of the eye and mucopurulent discharge. Conjunctival culture negative. Mother was negative for CT during pregnancy. Final clinical diagnosis: viral conjunctivitis.	Not compatible	Not typical presentation for CT
nCT-34	Adult male - Subacute bilateral ocular pain for 2 months. Diagnosis: anterior uveitis, first episode. Complete resolution with topical prednisolone.	Not compatible	Not typical presentation for CT / Improvement without treatment for CT
nCT-35	Neonatal - Conjunctivitis with bilareral purulent discharge. Culture of both conjunctiva positive for *Escherichia coli*. Mother negative for CT.	Not compatible	Alternative diagnosis available
nCT-36	16 year old female - Mucopurulent discharge, suspicion of viral conjunctivitis. Since patient was not verbal, many samples for cultures and NAAT taken during anesthesia for another reason. No follow up.	Not compatible	Not typical presentation for CT
nCT-37	Neonate female - Mother treated for both CT and NG 4 days before birth, cervical NAAT still positive at birth. Patient asymptomatic, no physical exam finding.	Indeterminate	
nCT-38	2 month old male - Bilateral conjunctival mucopurulent discharge, bronchiolitis, acute otitis media in a patient with recent URTI. Treated empirically with azythromycin for 2 days.	Not compatible	Not typical presentation for CT
nCT-39	Adult male - Chronic bilateral follicular conjunctivitis. Dry eyes, resolution with hydrating drops. Urine: CT NAAT negative.	Not compatible	Improvement without treatment for CT
nCT-40	Adult male - Papillary and purulent unilateral conjunctivitis. Diagnosis: papilloma with secondary canaliculitis with secondary recurrent conjunctivitis.	Not compatible	Not typical presentation for CT / Other diagnosis pathologically confirmed
nCT-41	Adult male - Blateral follicular conjunctivitis presumed to be caused by a virus. CT NAAT swab at follow up since persistant symptoms. Topical prednisone prescribed. Final diagnosis: idiopathic uveitis. Resolved at follow up.	Not compatible	Improvement without treatment for CT
nCT-42	Adult male - Post URTI (covid) bilateral chronic follicular conjunctivitis. Started right after the respiratory infection. Resolution on symptoms with topical antibiotics and artificial tears.	Not compatible	Improvement without treatment for CT

nCT-43	Adult male - Subacute keratoconjunctivitis for 10 days. Moxifloxacin and ciproflocacin drops prescribed. At follow-up, dendrites seen with fluorescine. Clinical HSV diagnosis, valacyclovir prescribed.	Not compatible	Not typical presentation for CT / Alternative diagnosis available
nCT-44	20 day old neonate - Mild discharge from right eye for 1 day. Mildly red conjunctiva, and mild superior eyelid edema. Treated with topical erythromycin, no follow up.	Not compatible	Improvement without treatment for CT
nCT-45	Neonate - Mild erythema and discharge at day 1. Mother negative for CT/NG during pregnancy. Topical erythromycin prescribed. Complete resolution 2 days later. Conjunctival culture negative.	Not compatible	Not typical presentation for CT / Improvement without treatment for CT
nCT-46	12 day old neonate - Mild discharge and erythema of left eye. Topical erythromycin prescribed, only applied once because of irritation. Spontaneously improved, but some secretions remaining. Treated with topical moxifloxacin for 2 weeks. Resolved at follow up.	Not compatible	Not typical presentation for CT / Improvement without treatment for CT
nCT-47	12-day-old neonate - Unilateral discharge with slight palpebral edema. Empirically prescribed oral azithromycin and topical erythromycin. No follicles on examination. Bacterial conjunctivitis was most probable diagnosis. Complete resolution at follow up.	Potentially compatible presentation	
nCT-48	Adult male - Chronic follicular HSV conjunctivitis. Was better with valacyclovir but when lowered the dose, conjunctivitis flared up. Improved with valacyclovir, but relapsed with decreasing dosage. With increased valacyclovir dosage, symptoms resolved.	Not compatible	Improvement without treatment for CT
nCT-49	Adult female - Keatoconjunctivitis initially treated with moxifloxacin drops: complete resolution. Symptoms reappeared 2 weeks later. Topical prednisolone and moxifloxacin prescribed: symptom resolved. Final diagnosis: Viral conjunctivitis. No systemic treatment given.	Not compatible	Improvement without treatment for CT
nCT-50	Adult male - Chronic bilateral follicular conjunctivitis. No response to loteprednol drops or valacyclovir. Prescription of fluorometholone drops. Resolution of symptoms.	Not compatible	Improvement without treatment for CT
nCT-51	17 year old female - Follicular post-viral conjunctivitis. Topical prednisolone prescribed, which completely resolved the symptoms. No antibiotics prescribed.	Not compatible	Improvement without treatment for CT

nCT-52	Adult female - Bilateral viral follicular conjunctivitis. Daughter of patient had conjunctivitis a few days earlier. Almost complete resolution with topical prednisone. No antibiotics prescribed.	Not compatible	Not typical presentation for CT / Improvement without treatment for CT
nCT-53	Adult male - Acute eye erythema and discharge. Physical examination positive for pre-auricular adenopathy. Conjunctival culture positive for Group A Streptococci. Resolution with oral amoxicillin and topical moxifloxacin.	Not compatible	Not typical presentation for CT / Improvement without treatment for CT / Other diagnosis microbiologically confirmed

#: arbitrary numbering system; nCT: negative for Chlamydia trachomatis; NG: *Neisseria gonorrhoeae*; NAAT: Nucleic Acid Amplification Test; STI: Sexually Transmissible Infection; TMP-SMX: Trimethoprime-Sulfamethoxazole; MALT: Mucosa Associated Lymphoid Tissue; HSV: Herpes simplex virus
