# Supplementary material for: Diagnosis of chlamydial and gonococcal conjunctivitis: performance evaluation of a nucleic acid amplification test
Source: Microbiol Spectr. 2026 Jul 10;14(8):e00701-26. doi: 10.1128/spectrum.00701-26 (PMC13436316; doi:10.1128/spectrum.00701-26)
Supplement: Supplementary file 3 [file spectrum.00701-26-s0003.pdf]

Table S3 - Clinical description for NG-negative samples

ID	Brief clinical description	Decision	Rationale if not compatible
nNG-01	Adult female - Presented with CT cervicitis with bilateral subacute follicular conjunctivitis with purulent discharge. Conjunctival samples positive for CT. Both cervicitis and conjunctivitis were diagnosed with NAAT. Received ceftriaxone and doxycyclin empirically. Resolution of symptoms.	Not compatible	Not typical presentation for NG
nNG-02	Adult female - Presented with chronic unilateral conjunctivitis. Improved with topical corticosteroids. Final diagnosis: papilloma which was subsequently removed. Complete resolution thereafter.	Not compatible	Not typical presentation for NG / Improvement without treatment for NG
nNG-03	1 month old - Crusting and discharge of left eye. Blepharoconjunctivitis that initially started with a rash on the patient's face. No discharge. Topical erythromycin resolved the issue. Mother negative for CT/NG.	Not compatible	Not typical presentation for NG
nNG-04	Adult male - Ocular globe trauma with rupture. No sign of infection. Prophylactic antibiotics prescribed.	Not compatible	Not typical presentation for NG
nNG-05	Adult female - Chronic unilateral papillary conjunctivitis. No discharge. Biopsy: chronic inflammation. Final diagnosis: dry eyes, artificial tears prescribed.	Not compatible	Not typical presentation for NG
nNG-06	Adult female - Hyperacute unilateral follicular and mucopurulent conjunctivitis. Empirical treatment with ceftriaxone and azithromycin. Culture negative. STI screening negative 1 month prior.	Potentially compatible	
nNG-07	13 year old female - Subacute conjunctival injection, no discharge. Most probable diagnosis: allergic conjunctivitis. Anti-histamines prescribed (systemic and topical), along with fluorometholone drops. Resolution of symptoms within 10 days.	Not compatible	Not typical presentation for NG / Improvement without treatment for NG
nNG-08	Neonate female - Screening in asymptomatic neonate. Mother had CT+ result during pregnancy, was treated 2 weeks prior to delivery. Cesarian section delivery.	Not compatible	Not typical presentation for NG
nNG-09	Adult male - Subacute bilateral ocular pain on/off for 2 months. Diagnosis: anterior uveitis, first episode. Complete resolution with topical prednisone.	Not compatible	Not typical presentation for NG / Improvement without treatment for NG
nNG-10	16 year old female - Keratoconjunctivitis initially treated with moxifloxacin drops, complete resolution. Symptoms reappeared 2 weeks later. Topical prednisolone and moxifloxacin prescribed: symptoms resolution. Final diagnosis: viral conjunctivitis. No systemic treatment given.	Not compatible	Not typical presentation for NG / Improvement without treatment for NG

nNG-11	Adult female - Unilateral eye erythema and discharge for 6 months. Treated twice with empirical azithromycin with no improvement. Final diagnosis: floppy eyelid syndome with secondary chronic conjunctivitis. Hydrating drops prescribed.	Not compatible	Not typical presentation for NG / Improvement without treatment for NG
nNG-12	Adult male - Post-URTI follicular conjunctivitis. Complete resolution with topical prednisolone. No systemic antibiotics prescribed.	Not compatible	Not typical presentation for NG / Improvement without treatment for NG

#: arbitrary numbering system; nNG: negative for *Neisseria gonorrhoeae*; CT: *Chlamydia trachomatis*; NG: *Neisseria gonorrhoeae;* NAAT: Nucleic acid amplification test; STI: Sexually transmitted infection; URTI: upper respiratory tract
